# An asparagine metabolism-based classification reveals the metabolic and immune heterogeneity of hepatocellular carcinoma

**DOI:** 10.1186/s12920-022-01380-z

**Published:** 2022-10-25

**Authors:** Jianguo Bai, Ruifeng Tang, Keyu Zhou, Jialei Chang, Hongyue Wang, Qixin Zhang, Jiahui Shi, Chao Sun

**Affiliations:** Department of Hepatobiliary Surgery, The Fourth Hospital of Hebei Medical University, Shijiazhuang, China

**Keywords:** Hepatocellular carcinoma, Asparagine metabolism, Tumor microenvironment, DNA damage response, GOT2

## Abstract

**Introduction and objectives:**

hepatocellular carcinoma (HCC) is the major form of liver cancer with a poor prognosis. Amino acid metabolism has been found to alter in cancers and contributes to malignant progression. However, the asparagine metabolism status and relevant mechanism in HCC were barely understood.

**Methods:**

By conducting consensus clustering and the least absolute shrinkage and selection operator regression of HCC samples from three cohorts, we classified the HCC patients into two subtypes based on asparagine metabolism level. The Gene Ontology, Kyoto Encyclopedia of Genes and Genomes analyses and Gene Set Enrichment Analysis of the differentially expressed genes between two subgroups were conducted. Immune cell infiltration was evaluated using CIBERSORT algorithm. The prognostic values of genes were analyzed by univariate and multivariate cox regression, ROC curve and Kaplan–Meier survival estimate analyses. Cell types of sing-cell RNA sequencing (scRNA-seq) data were clustered utilizing UMAP method.

**Results:**

HCC patients with higher asparagine metabolism level have worse prognoses. Moreover, we found the distinct energy metabolism patterns, DNA damage response (DDR) pathway activating levels, drug sensitivities to DDR inhibitors, immune cell compositions in the tumor microenvironment and responses to immune therapy between two subgroups. Further, we identified a potential target gene, glutamic-oxaloacetic transaminase 2 (GOT2). GOT2 downregulation was associated with worse HCC prognosis and increased infiltration of T regulatory cells (Tregs). ScRNA-seq revealed the GOT2 downregulation in cancer stem cells compared with HCC cells.

**Conclusions:**

Taken together, HCC subtype which is more reliant on asparagine and glutamine metabolism has a worse prognosis, and a core gene of asparagine metabolism GOT2 is a potential prognostic marker and therapeutic target of HCC. Our study promotes the precision therapy of HCC and may improve patient outcomes.

**Supplementary Information:**

The online version contains supplementary material available at 10.1186/s12920-022-01380-z.

## Introduction

Hepatocellular carcinoma (HCC) accounts for 90% of primary liver cancer. Its aggressive clinical behavior and few effective therapeutic options induce a poor prognosis [1]. Sorafenib had once been the only systemic therapy option for a decade, before the application of tyrosine kinase inhibitors, monoclonal antibodies against vascular endothelial growth factor receptor-2 (VEGF-2) and immune checkpoint blockers (ICBs) [2, 3]. The combination of atezolizumab with bevacizumab is a new reference standard in frontline systemic treatment for HCC [4]. However, the response rate of HCC patients is only one-third [5], which is even twice as the programmed death 1 (PD-1) monotherapy [6, 7]. Low response rate and lack of treatment guiding biomarkers are the main issues remaining unsolved. Better therapeutic strategies and biomarkers are still in urgent need.

Amino acids (AAs) build protein, sustaining cell proliferation. Cancer cells are mostly characterized by aberrated amino acid metabolism. Due to the increased proliferation rate, cancer cells need more nutrients to supply their hasty energy production and rushed protein synthesis processes [8]. Targeting amino acid acquisition and utilization is a promising therapeutic strategy. Aspartate is a significant metabolic hub and is required for the synthesis of purines and pyrimidines [8]. Tumors rely on aspartate for continued growth in hypoxic environments [9, 10]. Asparagine synthetase (ASNS) catalyzes the synthesis of the non-essential amino acid asparagine from aspartate. Acute lymphoblastic leukemia (ALL) barely expresses ASNS and is thus sensitive to asparagine depletion therapy by asparaginase (ASNase). ASNase has also greatly improved the outcome of NK/T cell lymphoma (NKTCL) [11]. Despite the successful application in ALL and NKTCL patients, ASNase has not been proved to be effective in many other cancers, due to their reduced dependency on circulating asparagine [12]. Tumors with elevated ASNS activity may acquire proliferation advantage and chemotherapy resistance. Pancreatic ductal carcinomas with higher ASNS expression are more resistant to cisplatin [13]. Upregulated ASNS expression was also observed in gastric cancer compared with normal tissue, which predicts worse survival outcomes [14]. ASNS expression is an independent prognostic factor of HCC [15], even though the mechanism of ASNS maintaining the cellular homeostasis remains largely unknown. ASNase treatment is therapeutically explorable, with its molecular mechanism under investigated [12].

Asparagine has been found to regulate tumorigeneses signaling pathways. Asparagine activates the mechanistic target of rapamycin complex 1 (mTORC1) and the activating transcription factor 4 (ATF4) in tumor cells in response to mitochondrial electron transport chain (ETC) inhibition [16]. Moreover, asparagine inhibits the AMP-activated protein kinase (AMPK) by directly binding to its upstream suppressor LKB1 [17]. However, the biological significance of asparagine regulating these pathways is still unclear. Whether or how this is related to protein synthesis, nucleotide synthesis and energy supply needs further investigation. Interestingly, asparagine enhances the T cell receptor (TCR) signaling to promote CD8 + T cell activation and its anti-tumor responses [18].

The asparagine and glutamine metabolism are undetachable. Glutamine is often found limited in the tumor microenvironment, due to preferentially depletion by tumor [19]. Tumors tend to employ adaptive mechanisms to maintain cellular glutamine level. Blocking cellular glutamine intake or inhibiting glutamine metabolism enzymes are promising therapeutic strategies [20]. Inhibiting glutaminase function are preclinically effective for non-small cell lung cancer [21]. Extracellular supplementation of asparagine can support tumor cell survival when exogenous glutamine is depleted [19]. Targeting asparagine bioavailability can prevent tumor cells from adapting to the glutamine lacking environment [19]. Asparagine directly supports protein synthesis under glutamine-deprivation and promotes epithelial-mesenchymal transition (EMT) to initiate tumor metastasis [19, 22]. Asparagine restriction limits the EMT process [22]. The metabolomics study revealed that HCC has altered aspartate metabolism [23]. However, the exact mechanism of its cause and influence has been poorly understood.

Previous studies have demonstrated that some specific asparagine metabolism genes are critical for HCC progression and prognosis. For example, ASNS is an independent prognostic factor of HCC [15]. Moreover, SLC25A12 upregulation promotes the growth of HCC cells [24]. In addition, SLC25A13 gene mutations may concern the susceptibility of HCC [25]. Nonetheless, the holistic status of asparagine metabolism in HCC has not been clearly depicted. Here we utilized the bioinformatics methods to investigate the asparagine metabolism of HCC, seeking to identify new biomarkers for HCC prognosis and targets for clinical treatment.

## Materials and methods

### Datasets

The RNA-sequencing expression profiles and corresponding overall survival (OS) and disease-free survival (DFS) data of HCC were acquired from the Cancer Genome Atlas (TCGA), International Cancer Genome Consortium (ICGC) database and GSE84598 dataset in the Gene Expression Omnibus (GEO) database [26–29]. A total of 371 HCC patients was retrieved from the TCGA database. The inclusion criteria of TCGA were patients that were diagnosed with HCC, and had not received prior treatment for their disease (ablation, chemotherapy, or radiotherapy). Also, 240 primary HCC cases contributed by Institute of Physical and Chemical Research (RIKEN) in the ICGC database (project code: LIRI-JP) were retrieved. The GSE84598 dataset contained 22 confirmed HCC cases undergoing resection at the Department of Surgery, University of Mainz, Germany. The single-cell RNA sequencing (scRNA-Seq) data of HCC were acquired from GSM3064824 sample in the GEO database [30]. The samples with no missing variables information were analysed, and if the selected variable is missing, the sample is deleted.

### Prognostic signature construction

We acquired the human asparagine metabolism gene set “REACTOME_ASPARTATE_AND_ASPARAGINE_METABOLISM” from Reactome (identifier: R-HSA-8963693) [31, 32]. Then we conducted consensus clustering using R software to identify subtypes of HCC in TCGA database and GSE84598 dataset based on the expression pattern of asparagine metabolism gene set. Cluster map was plotted using R. The least absolute shrinkage and selection operator (LASSO) regression algorithm was performed for the HCC samples in the ICGC database using R [33]. To analyse the OS and/or DFS of different subgroups, p-values and hazard ratio (HR) with 95% confidence interval (CI) were generated by log-rank tests and univariate cox proportional hazards regression. Time ROC analysis was conducted by R package to investigate the predictive accuracy of the gene set and the risk score.

### Identification of differentially expressed genes (DEGs) and functional analyses

The DEGs between HCC subgroups were assessed utilizing R. The threshold for differential mRNA expression was “P-adjust < 0.05 and |fold change|> 2”. The volcano plot and heatmap were constructed using the fold change values and P-adjust. Gene Ontology (GO) term and Kyoto Encyclopedia of Genes and Genomes (KEGG) pathway analyses were conducted using R [34–37]. Gene Set Enrichment Analysis (GSEA) analysis was conducted with GSEA software (V4.1.0).

### Chemotherapeutic response prediction

We predicted the half-maximal inhibitory concentration (IC50) of each HCC sample in the TCGA database to different drugs based on the Genomics of Drug Sensitivity in Cancer (GDSC) database [38]. The prediction was implemented by R.

### Immune cell infiltration analysis and prediction of immunotherapy response

We analysed the immune cell infiltration in the two subgroups of HCC in TCGA database by CIBERSORT algorithm [39]. The heatmap of infiltrating immune cells’ percentage in each sample were plotted by R. Potential responses to immune checkpoint blockade (ICB) therapy of the two HCC subgroups were predicted with TIDE algorithm and were plotted using R [40]. The association between GOT2 expression and the infiltration of Treg cells of HCC in the TCGA database was analysed by CIBERSORT algorithm [39], CIBERSORT-ABS [41] and QUANTISEQ [42] algorithm. The correlation between the expression of GOT2 and eight immune checkpoint genes were plotted by R.

### Evaluation of the prognostic value of asparagine metabolism related genes

Univariate and multivariate cox regression analyses of the genes involved in asparagine metabolism were performed using R. A nomogram was developed based on the results of multivariate cox proportional hazards analyses to predict the 1-, 3- and 5-year overall recurrence by R. The OS, progression-free interval (PFI) and disease-specific survival (DSS) analyses of HCC patients with high or low GOT2 expression in the TCGA database were conducted by R. To estimate the diagnostic value of GOT2, we performed the ROC analysis using R software [43]. Also, we used KM Plotter (http://kmplot.com) website tool to analyze whether the prognostic value of GOT2 was associated with Treg infiltration in HCC [44]. A total of 371 HCC samples from TCGA database was stratified into two groups, Treg enriched or decreased. Then we analyzed the OS and recurrence-free survival (RFS) of high and low GOT2 cases separated by the median expression in each group.

### ScRNA-seq data analysis

We re-annotated the cell cluster of GSM3064824 sample by UMAP method, which was different from the t-SNE method in the original report [30]. We performed cell clustering using the FindClusters function by R. The marker gene for cell typing were described previously [45–47]. Then we evaluated the GOT2 expression in HCC cell and cancer stem cell.

### Statistical analysis

Data were expressed as mean ± SD. Analyses were performed in GraphPad Prism (version 8.0) or R software (v3.4.4). P < 0.05 were considered statistically significant.

## Results

### Asparagine metabolism-based subclasses of HCC in TCGA and ICGC database

**Fig. 1:**
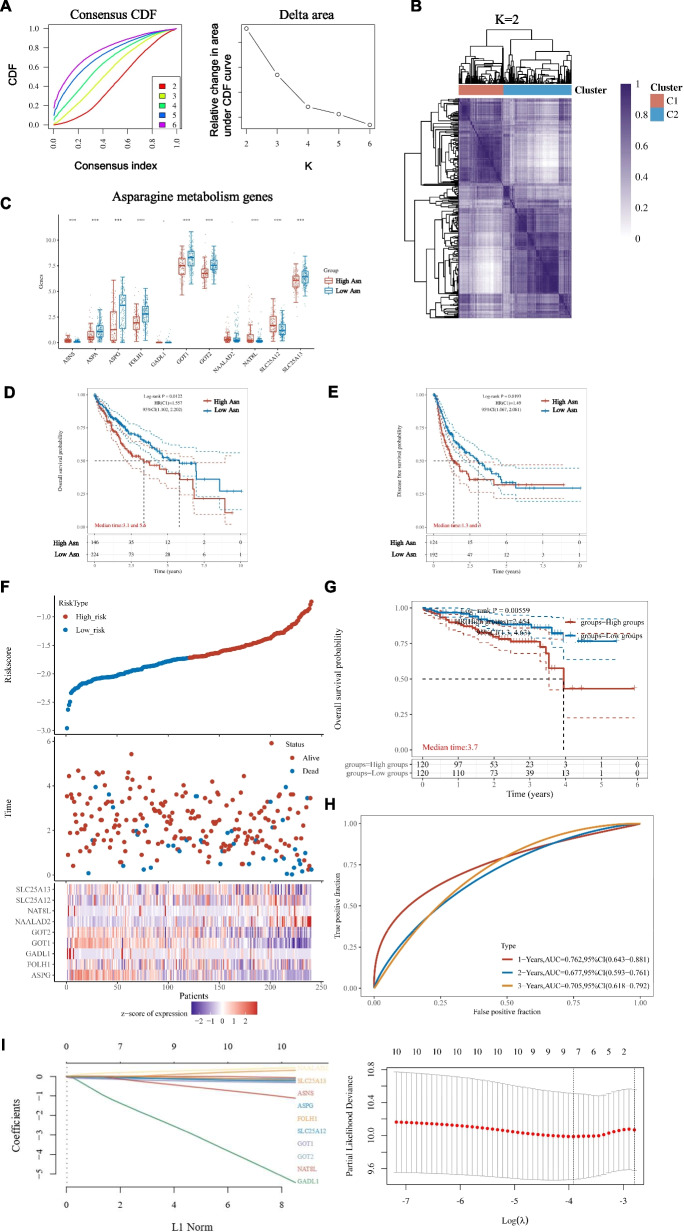
Identify asparagine metabolism subtypes of HCC in TCGA database and validation in ICGC database. **A** Consensus Cumulative Distribution Function (CDF) Plot and relative change in the area under the CDF curve (CDF Delta area). **B** Consensus matrices of the TCGA cohort for k = 2. **C** The expression of asparagine metabolism genes in two HCC subgroups. **D**, **E** OS and DFS analyses of the two HCC subgroups in the TCGA database. **F** A 9-gene signature was constructed for the HCC cases in the ICGC database by Lasso Cox analysis. Risk scores distribution, survival status of each patient in the ICGC database, and heatmaps of signature gene expression were plotted. **G** OS analysis of the two HCC subgroups in the ICGC database. **H** The 1-, 3- and 5-year ROC curves of the gene signature. The AUC was indicated. **I** The relationship between partial likelihood deviation and log (λ), and the LASSO coefficient profiles of the fractions of 9 genes were plotted

The asparagine metabolism gene set consists of ASNS, ASPA, ASPG, FOLH1, GADL1, GOT1, GOT2, NAALAD2, NAT8L, SLC25A12 and SLC25A13. The interaction between proteins of these genes were shown by PPI network (Additional file 1: Fig. S1). Based on the expression pattern of asparagine metabolism genes, we classified the HCC patients in the TCGA database into two subtypes after performing k-means clustering (Fig. 1A, B). Group 1 was defined as the high asparagine metabolism subgroup, as characterized by higher expression of asparagine synthesis gene ASNS, and lower expression of asparagine degradation gene ASPG compared with Group 2, the low asparagine metabolism group (Fig. 1C). Moreover, GOT2 was lower in Group 1. GOT2 transits oxaloacetate and glutamate to aspartate and α-ketoglutaric acid (αKG), and vice versa [48]. That indicated less active tricarboxylic acid (TCA) cycle and more active protein synthesis using asparagine in Group 1, the high asparagine metabolism subgroup (Additional file 2: Fig. S2). High asparagine metabolism subgroup has worse OS (3.1 VS 5.6 years) and worse DFS (1.3 VS 3 years) than low asparagine metabolism subgroup (Fig. 1D, E). The characteristics of HCC cases in the TCGA database were described (Additional file 4: Table S1).

We further validated the asparagine metabolism-based classification utilizing the HCC samples in the ICGC database. A signature of 9 genes related with asparagine metabolism were developed for the prediction model in the LASSO regression analysis. The correlation among risk score, survival status and the signature gene expression were plotted, revealing the association between high signature risk score and worse survival status (Fig. 1F). The OS of high asparagine metabolism subgroup was significantly worse than low asparagine metabolism subgroup, as shown by the KM analysis (Fig. 1G). The ROC curve of the gene signature was plotted, and the area under the curve (AUC) values for 1-, 2- and 3- year ROC curves were 0.762, 0.677 and 0.705, respectively (Fig. 1H). The risk score model was developed on the algorithm: Risk score = (− 0.0963) × ASPG + (− 2e − 04) × FOLH1 + (− 1.8193) × GADL1 + (− 0.1856) × GOT1 + (− 0.0683) × GOT2 + (0.2245) × NAALAD2 + (− 0.254) × NAT8L + (− 0.0721) × SLC25A12 + (0.0152) × SLC25A13, lambda.min = 0.0199 (Fig. 1I).

### DEGs between subgroups and function analyses

**Fig. 2:**
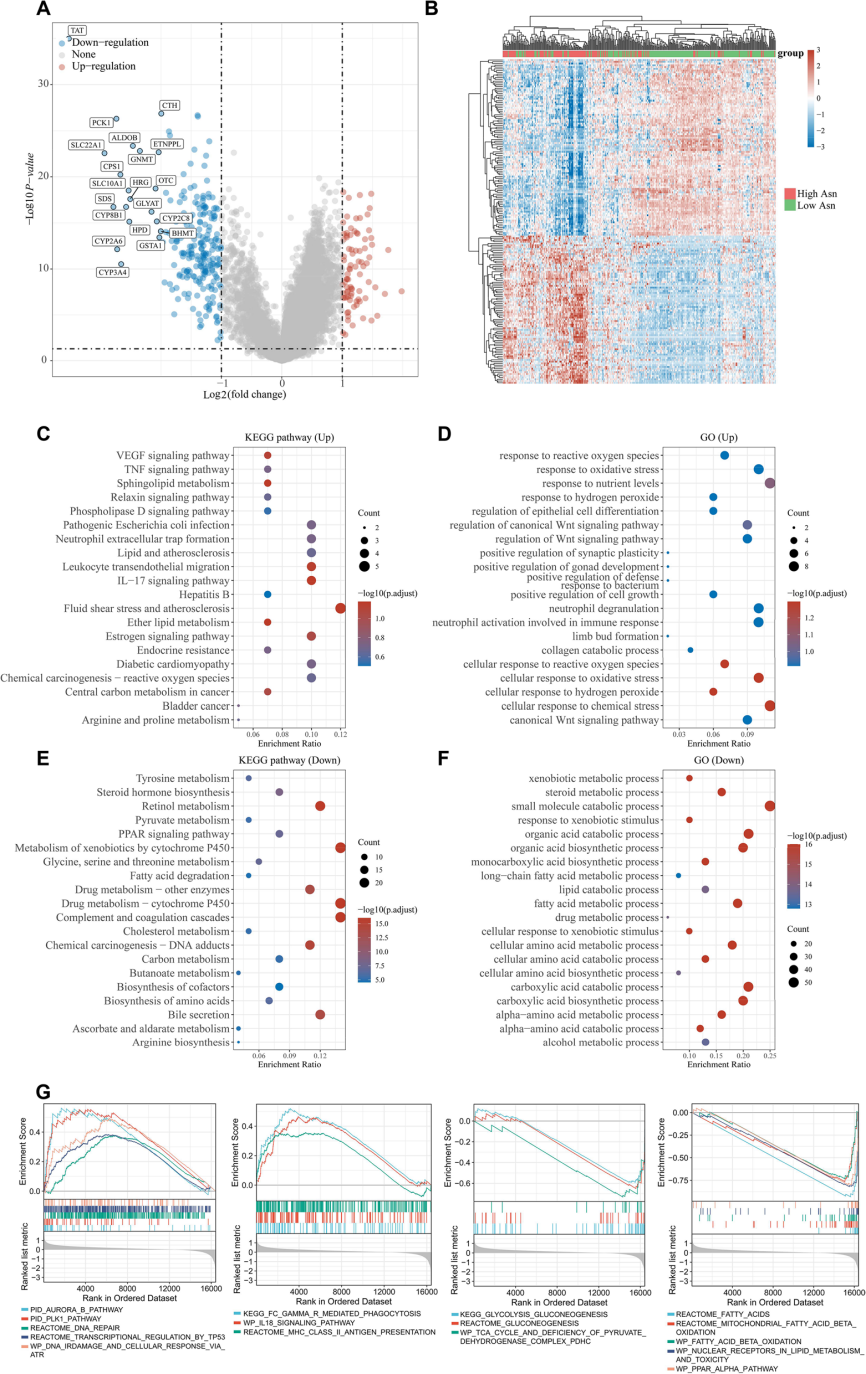
Identification of differentially expressed genes (DEGs) between two subgroups in the TCGA database. **A** Volcano plot of the DEGs between high and low asparagine metabolism subgroups in the TCGA database. The threshold was set as |log2 Fold change|> 1 and p < 0.05. **B** Heatmap shows the gene expression profile in high and low asparagine metabolism subgroups. **C**, **D** Up-regulated GO and KEGG terms of the DEGs. **E**, **F** Down-regulated GO and KEGG terms of the DEGs. **G** GSEA analysis of the DEGs

To better understand the mechanisms causing prognosis difference between HCC subgroups, we investigated the DEGs between two subgroups in TCGA database (Fig. 2A, B). GO and KEGG analyses of the DEGs revealed the upregulated VEGF signaling pathway, TNF signaling pathway, IL-17 signaling pathway, response to reactive oxygen species, response to nutrient levels, regulation of Wnt signaling pathway, positive regulation of cell growth in high asparagine metabolism subgroup, compared with low asparagine metabolism subgroup (Fig. 2C, D). While the steroid hormone biosynthesis, PPAR signaling pathway, metabolism of xenobiotics by cytochrome P450, drug metabolism, small molecule catabolic process, organic acid catabolic and fatty acid metabolic process were downregulated in high asparagine metabolism subgroup, compared with low group (Fig. 2E, F).

To further explore the functions of the DEGs between two HCC subgroups, GSEA analysis was conducted. GSEA analysis results showed that DEGs mainly enriched in DNA damage response (DDR) pathway (including DNA repair, transcriptional regulation of TP53, DNA IR damage and cellular response via ATR, aurora B pathway, and PLK1 pathway), immune response (including Fc gamma R mediated phagocytosis, MHC II antigen presentation, IL-18 signaling pathway), glutamine metabolism (including glycolysis/gluconeogenesis, TCA cycle and deficiency of pyruvate dehydroge), and fatty acid metabolism (including fatty acid, mitochondrial fatty acid beta oxidation, fatty acid beta oxidation, nuclear receptors in lipid metabolism and toxic, and PPAR alpha pathway) (Fig. 2G).

### Metabolic characteristics of HCC subclasses

**Fig. 3:**
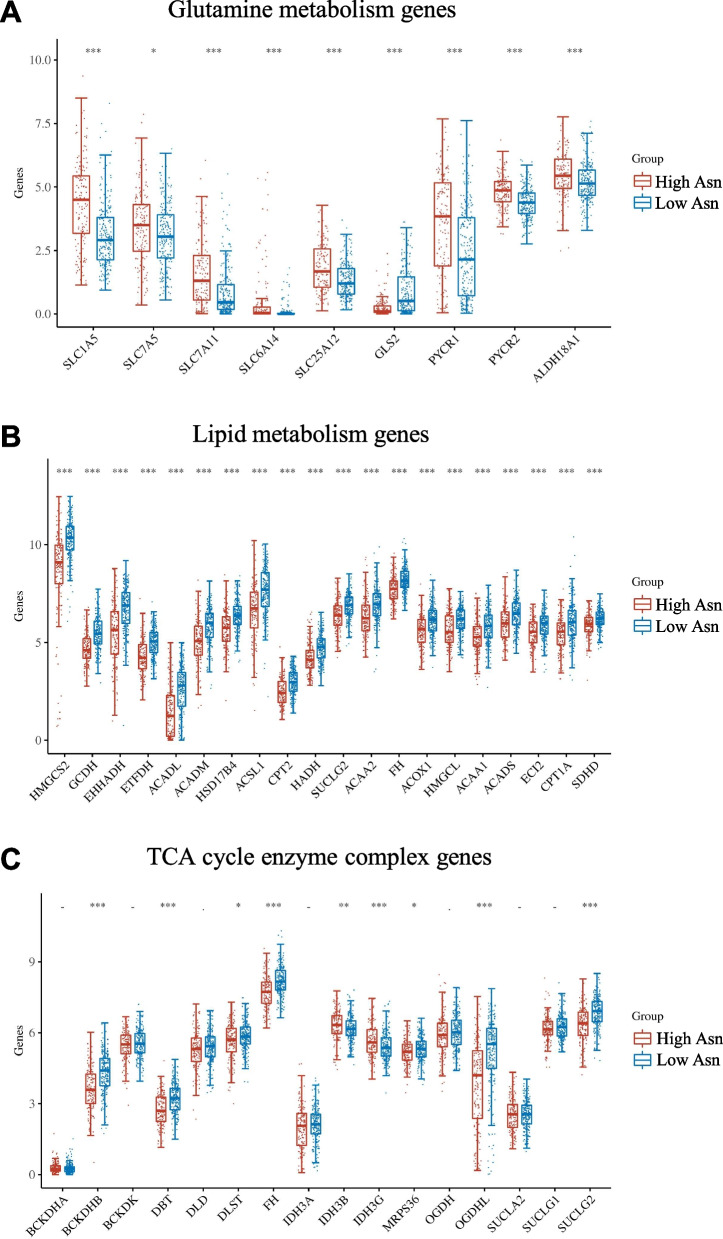
Expression comparison of metabolic-related genes between two HCC subgroups in the TCGA database. Glutamine metabolism genes (**A**), lipid metabolism genes (**B**) and TCA cycle enzyme complex genes (**C**) were acquired from Wikipathway, the expression of which in HCC subgroups were plotted. *p < 0.05; **p < 0.01; ***p < 0.001

As indicated by the function analyses, energy metabolism differs in the two HCC subgroups. We extracted the core genes’ expressions involved in glutamine metabolism, glucose aerobic oxidation and fatty acid metabolism of HCC samples in the TCGA database. The core genes of glutamine metabolism, for example, ASCT2, were upregulated in high asparagine metabolism HCC subgroup (Fig. 3A; Additional file 2: Fig. S2). The GLS2 gene was responsible for catalyzing the glutamine into glutamate, which was downregulated in high asparagine metabolism subgroup (Fig. 3A; Additional file 2: Fig. S2). While the genes concerning fatty acid metabolism and TCA cycle enzyme complex were downregulated in high asparagine metabolism HCC subgroup (Fig. 3B, C). These results indicated an energy resource preference shift from glucose and fatty acid to asparagine and glutamine in the HCC subgroup with higher asparagine metabolism level and worse prognosis.

### Validation of asparagine metabolism-based classification of HCC in the GEO database

**Fig. 4:**
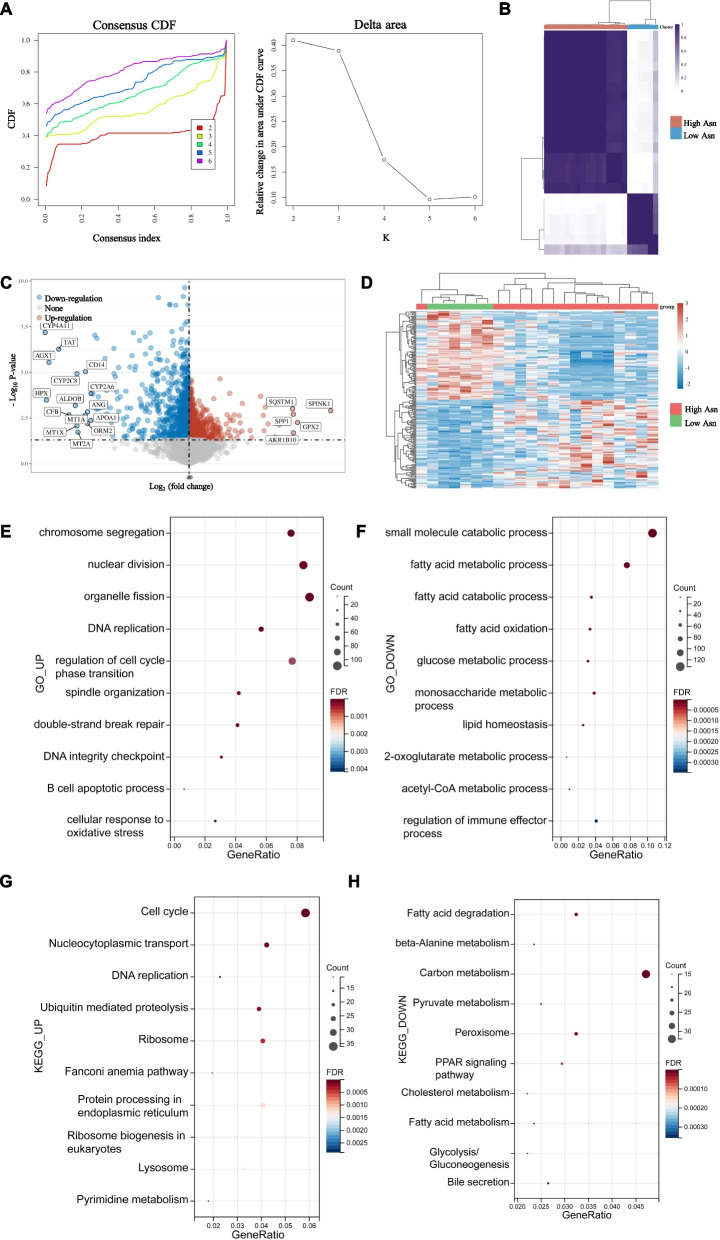
Signature validation using GSE84598 dataset in the GEO database and subgroup characteristics comparison. **A** The cumulative distribution function (CDF) curve and the delta area curve of consensus clustering. **B** Consistency of clustering results heatmap (k = 2). **C** Volcano plot of the DEGs between two HCC subgroups in the GSE84598 dataset. The threshold was set as |log2 Fold change|> 1 and p < 0.05. **D** Gene expression profile heatmap of two subgroups. **E**–**H** GO and KEGG analyses of DEGs

To further validate the energy metabolism pattern shift between HCC subgroups separated by asparagine metabolism levels, we classified the HCC patients in GSE84598 dataset into two subgroups based on the expression pattern of asparagine metabolism genes (Fig. 4A, B). Further we analyzed the DEGs between two subgroups (Fig. 4C, D). The DEGs upregulated in high asparagine metabolism subgroup were involved in GO biological processes including organelle fission, nuclear division, chromosome segregation and double-strand break repair, etc. (Fig. 4E). On the contrary, the small molecule catabolic process, fatty acid metabolic process, glucose metabolic process, 2-oxoglutarate metabolic process, acetyl-CoA metabolic process and regulation of immune effector process were downregulated in high asparagine metabolism subgroup (Fig. 4F). KEGG analysis revealed upregulated pathways including cell cycle, nucleocytoplasmic, DNA replication and ubiquitin mediated proteolysis in high asparagine metabolism subgroup (Fig. 4G). While the carbon metabolism, peroxisome, fatty acid degradation, PPAR signaling pathway and glycolysis/gluconeogenesis pathways were down-regulated in high asparagine metabolism subgroup (Fig. 4H). These results confirmed that HCC with active asparagine metabolism had a distinct energy resource with lowered glutamine and fatty acid consumption.

### Targeting DDR pathway in HCC

**Fig. 5:**
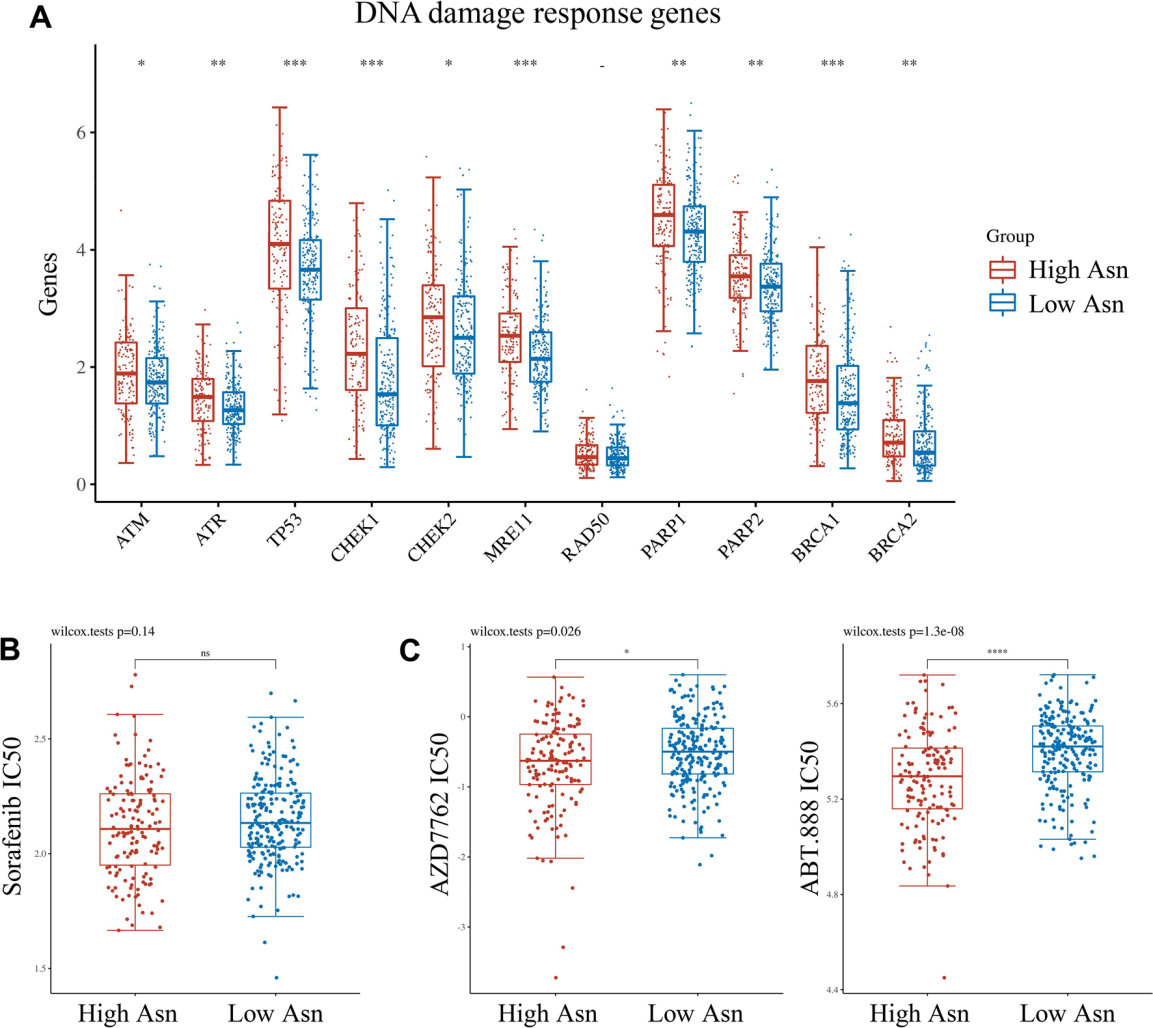
DNA damage response pathway in the two subgroups and relative drug sensitivity analyses of HCC in the TCGA database. **A** The expressions of representative genes of DNA damage response pathway in high asparagine metabolism HCC subgroup were significantly higher than the low asparagine metabolism group. **B** The IC50 of two subgroups in response to sorafenib showed no difference. **C** The IC50 of high asparagine metabolism subgroup in response to CHEK1 inhibitor AZD7762 and PARP 1/2 inhibitor ABT-888 were lower than the low asparagine metabolism subgroup

The aberration of DDR pathway contributes to tumorigenesis. Lots of DNA repair molecules are potential antitumor targets. Since DNA damage and repair related processes and pathways were enriched in the DEGs between two HCC subgroups, we further evaluated 11 core molecules of DNA repair process to determine their potential value as treatment targets. The expression of ATM, ATR, TP53, CHEK1. CHEK2, MRE11, PARP1, PARP2, BRCA1, and BRCA2 were significantly higher in high asparagine metabolism subgroup of HCC (Fig. 5A). Sorafenib is one of the first line therapy of HCC, which shows no therapeutic effect difference on two subgroups (Fig. 5B). However, CHEK1 inhibitor AZD7762 and PARP 1/2 inhibitor ABT-888 were predicted to have better therapeutic effect on high-asparagine metabolism subgroup, as shown by the lower IC50 value (Fig. 5C). This indicated that the worse prognosis of high asparagine metabolism HCC subgroup under current treatment could be ameliorated by the DDR inhibitors.

### HCC subgroup with high level of asparagine metabolism has suppressive immune microenvironment

**Fig. 6:**
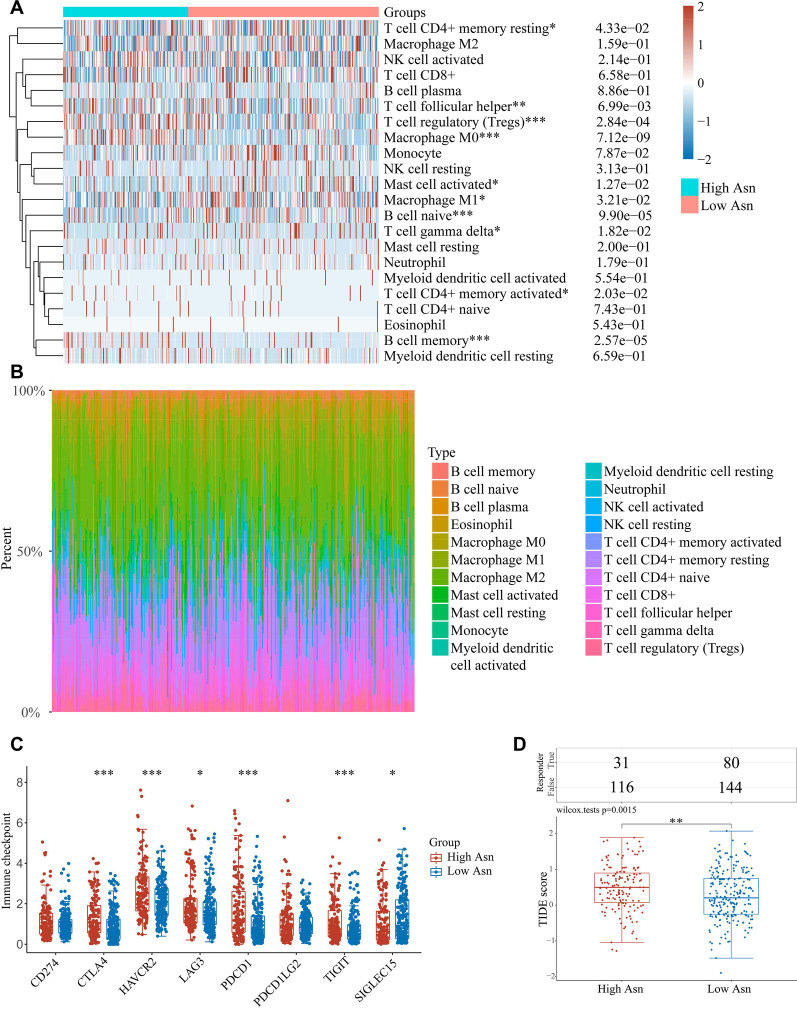
The immune landscape of high and low asparagine metabolism HCC subgroups in the TCGA database. **A** The immune cell infiltration in high and low asparagine metabolism subgroups. **B** Immune cell proportion in each HCC cases. **C** The expressions of most immune checkpoint molecules in high asparagine metabolism subgroup were significantly higher than the low asparagine metabolism group. **D** The TIDE score of the HCC cases predicted that high asparagine metabolism subgroup had better response to immune checkpoint blockers (ICBs) than low asparagine metabolism group

As implied by the function analyses of DEGs between HCC subgroups in TCGA database, immune response may alter in two subgroups. We analyzed the cell composition in the immune microenvironment of two HCC subgroups using the CIBERSORT algorithm. To our notice, the high-asparagine metabolism subgroup has more Treg cells, T follicular helper cells, M0 macrophage cells and memory B cells infiltration, but less resting memory CD4 + T cells, mast cell, M1 macrophage cells, naïve B cells and gamma delta T cells infiltration in immune microenvironment (Fig. 6A). The percentage of different kinds of immune cells in every sample was plotted (Fig. 6B).

Immune checkpoints and their ligands expressed on HCC cells or immune cells contribute to immune evasion. The immune checkpoint gene markers’ expressions (including CTLA4, HAVCR2, LAG3, PDCD1, TIGHT) were higher in high asparagine metabolism HCC subgroup (Fig. 6C). The expression levels of immune checkpoint genes are relevant to the tumor response to immune checkpoint blockade therapy. Consistently, high asparagine metabolism subgroup had higher TIDE score, which predicted a worse immune therapy response (Fig. 6D). More gene signatures that predict the ICI response of HCC patients were analyzed in two subgroups of HCC samples in TCGA (Additional file 3: Fig. S3; Additional file 6: Table S3).

### Prognostic value of asparagine metabolism genes

**Fig. 7:**
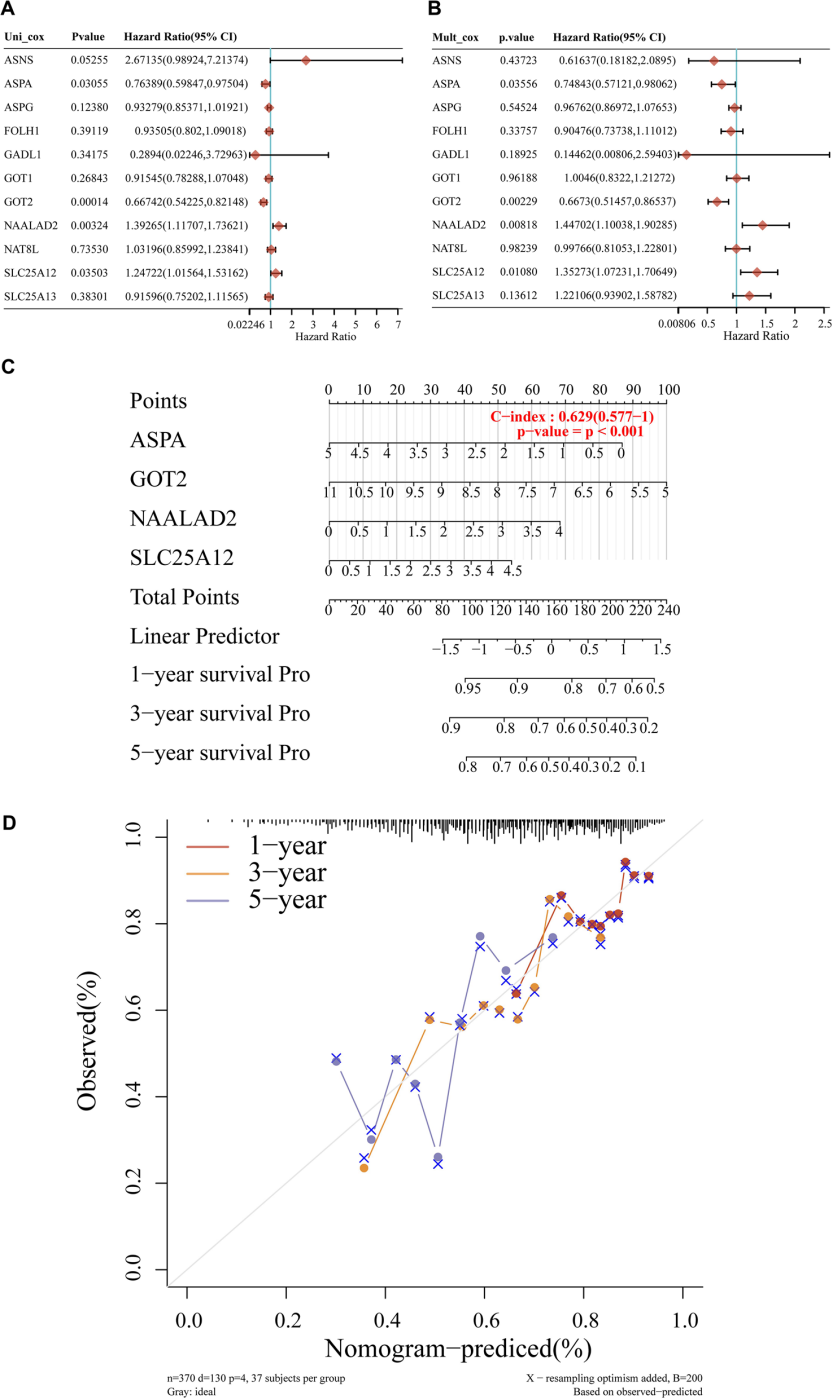
Evaluation of the prognostic value of every single gene in the asparagine metabolism gene set. **A**, **B** The univariate and multivariate Cox regression of genes involved in asparagine metabolism in term of OS. **C** Nomograms predicting the 1-, 2- and 3-year OS of HCC based on the expression of ASPA, GOT2, NAALAD2 and SLC25A12. **D** Calibration curve for the OS nomogram model

Given the distinct characteristics of two HCC subgroups classified by the expression of 11 asparagine metabolism genes, we seek to identify the most important gene concerning patients’ fate among the gene set. Univariate and multivariate Cox regression analyses revealed the prognostic value of the 11 asparagine metabolism genes in predicting the OS of HCC patients in TCGA database (Fig. 7A, B). GOT2 gene showed significant OS prediction ability (Fig. 7A, B). Nomogram provided the graphical risk calculation for the 1-, 3-, and 5- year OS of HCC patients, using the independent expressions of ASPA, GOT2, NAALAD2 and SLC25A12 gene (Fig. 7C). The predicting ability of the nomogram was visualized by the calibration curve (Fig. 7D).

### Low GOT2 expression predicts worse prognosis of HCC

**Fig. 8:**
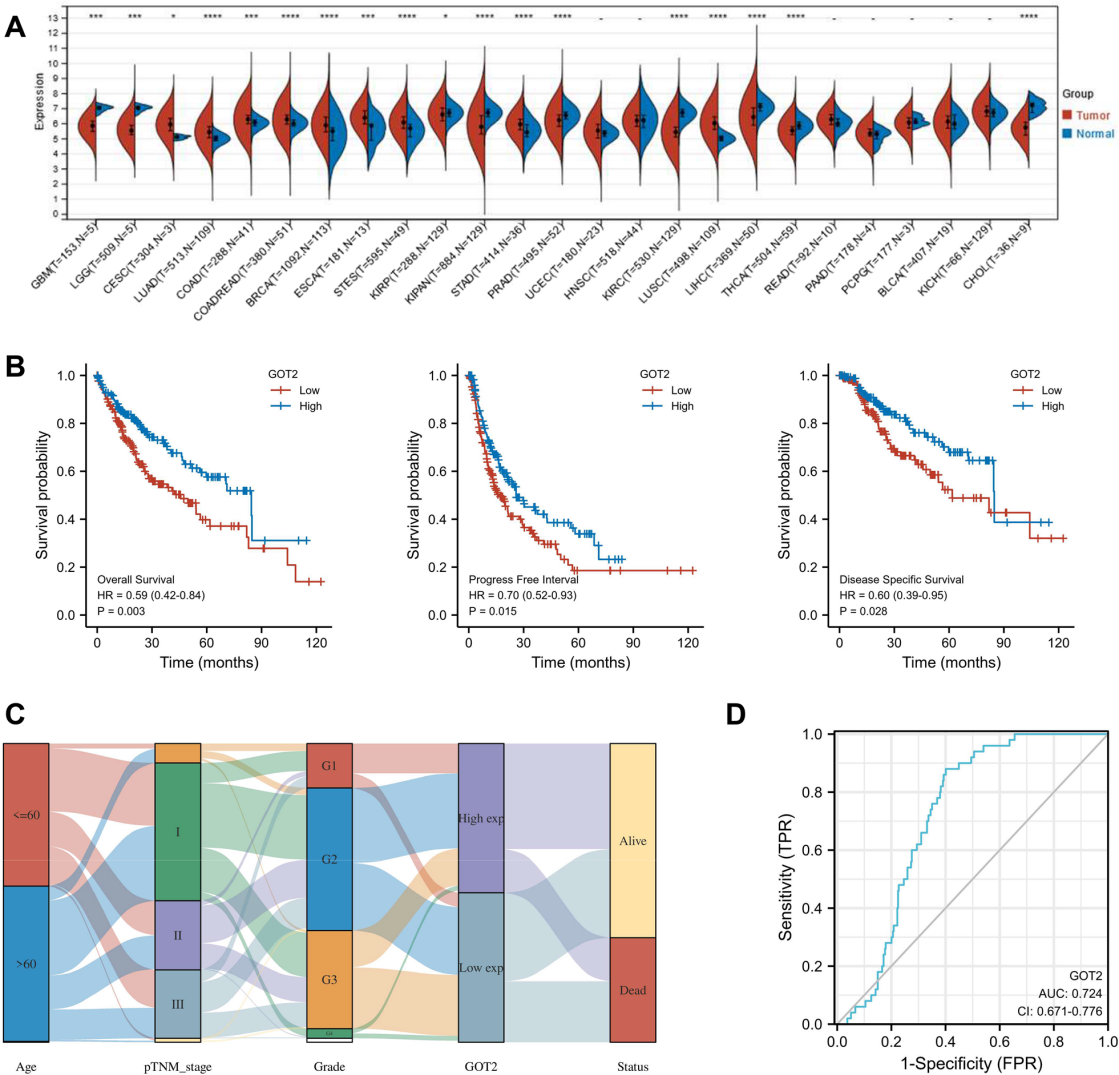
GOT2 down-expression predicts worse prognosis of HCC patients in the TCGA database. **A** The pan-cancer analysis of GOT2 expression. **B** The association between GOT2 expression and the OS, PFI, and DSS of HCC patients. **C** The Sankey diagram showing the distribution of age, pTNM stage, grade, GOT2 expression and survival status of HCC samples. **D** The ROC curve of GOT2 gene, with the AUC value indicated

Since GOT2 has profound prognostic value, and its function was poorly understood in HCC, we further investigated its expression and function. We first accessed the pan-cancer expressions of GOT2 in the TCGA database. GOT2 was downregulated in glioblastoma multiforme (GBM), brain lower-grade glioma (LGG), kidney renal papillary cell carcinoma (KIRP), pan-kidney cohort (KIPAN), prostate adenocarcinoma (PRAD), kidney renal clear cell carcinoma (KIRC), liver hepatocellular carcinoma (LIHC), thyroid carcinoma (THCA), cholangiocarcinoma (CHOL) (Fig. 8A). Low GOT2 level was correlated with worse OS, PFI, and DSS of HCC in the TCGA database (Fig. 8B). Sankey diagram visualized the variables’ distribution of every HCC sample including age, pTNM stage, grade, GOT2 expression and patient status (Fig. 8C). In addition, the patients’ information detached by GOT2 median expression were shown in Additional file 5: Table S2. The ROC curve evaluated the predictive performance of GOT2 (Fig. 8D). The AUC was 0.724 (Fig. 8D). These results confirmed the association between low GOT2 expression and HCC prognosis.

### The prognostic value of GOT2 relies on Treg abundance

**Fig. 9:**
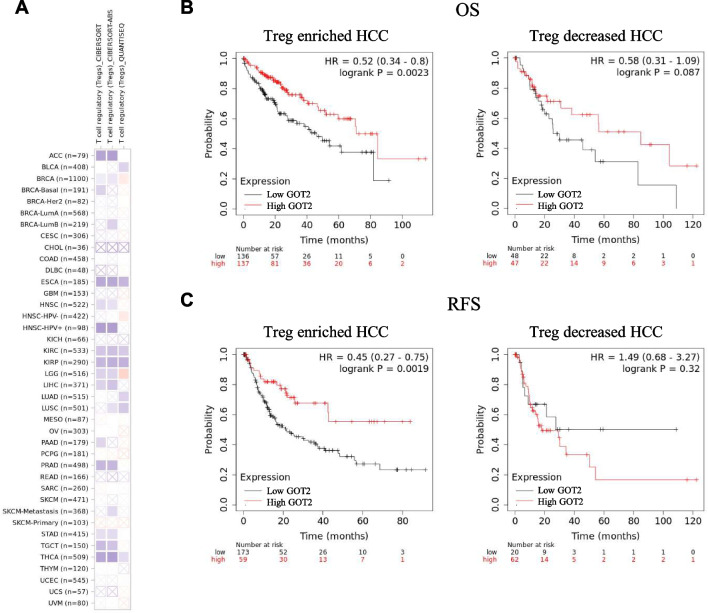
GOT2 expression and Treg cell infiltration. **A** Pan-cancer correlation analysis of the GOT2 expression and the infiltration of Treg cells. Correlation of GOT2 expression with OS (**B**) and RFS (**C**) in Treg enriched and decreased HCC subgroups

Treg cell modulates tumor immune evasion. We analyzed the pan-cancer correlation between GOT2 expression and Treg cell infiltration in the TCGA database. CIBERSORT, CIBERSORT-Abs and QUANTISEQ analyses showed that GOT2 expression was negatively related with Treg cell infiltration in HCC/LIHC, and many other cancers including ACC, ESCA, HNSC-HPV + , KIRC, KIRP, PRAD, STAD, TGCT, THCA, etc. (Fig. 9A). Then we investigated whether GOT2 exert influence on HCC prognosis through modulating Treg cell infiltration. We separated the HCC patients in the TCGA database into Treg enriched or decreased groups, and analyzed the OS and RFS of patients with high or low GOT2 expression in these two groups. We found that in Treg enriched cases, low GOT2 expression was associated with worse OS and RFS (Fig. 9B, C). The median OS for Treg enriched HCC patients with high or low GOT2 expression (divided by median expression) were 71.03 and 46.2 months, respectively (p = 0.0023). While in Treg decreased HCC patients, high or low GOT2 expression subgroups have the median OS of 84.73 and 25.6 months (p = 0.087). The RFS of the Treg enriched cases with high or low GOT2 expression were 21.3 and 7.97 months (p = 0.0019), while the Treg decreased cases were 9.1 and 6.5 months, respectively (p = 0.32). These results indicated that GOT2 was related with HCC prognosis only when Treg cells were abundant in the immune microenvironment. GOT2 may modulate Treg cell infiltration to shape the immune microenvironment and influence HCC prognosis.

### Single cell sequencing reveals GOT2 expression and differential pathways in HCC cells and cancer stem cells

**Fig. 10:**
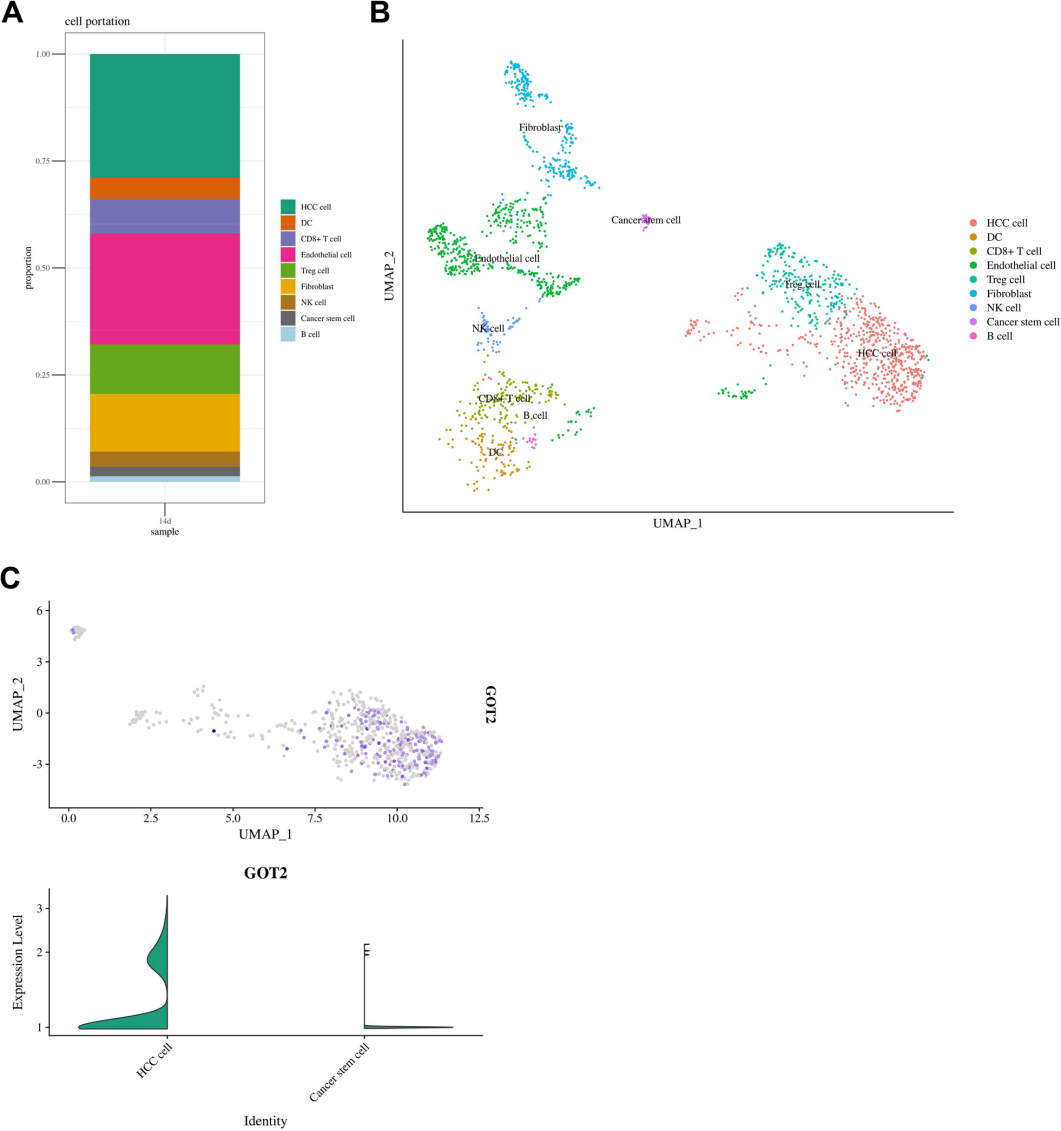
GOT2 expression and core pathway differences in HCC cells and cancer stem cells. **A**, **B** The cell proportion and cell type distribution of HCC tissue. **C** GOT 2 expressions in HCC cells and cancer stem cells

Upon analyzing the single cell sequencing data of a HCC sample GSM3064824 from the GEO database, we classified the cell population and identified HCC cell, DC, CD8^+^ T cell, endothelial cell, Treg cell, fibroblast, NK cell, cancer stem cell and B cell (Fig. 10A). Cell distribution was visualized (Fig. 10B). GOT2 expression was lower in cancer stem cells than in HCC cells (Fig. 10C).

## Discussion

Targeting asparagine metabolism is a promising antitumor strategy. Notably, we firstly establish a novel metabolic classifier for three HCC cohorts (TCGA, ICGC and GSE84598) based on asparagine metabolism. The HCC subgroup with higher asparagine metabolism level has elevated glutamine metabolism and down-regulated glucose aerobic oxidation and fatty acid metabolism, compared with the other subgroup with lower level of asparagine metabolism. GOT2 hypo-expression may contribute to the metabolism pattern shift and thus worsen the prognosis of HCC patients. The variations between two subgroups also include the DDR pathway activation and the infiltration of immunosuppressive cells (e.g., Treg cells). Higher asparagine metabolism group has overactivated DDR pathway, and are more sensitive to DDR inhibitors. Treg cells are more enriched in HCC with higher asparagine metabolism level, predicting worse responses to ICB therapy. Importantly, through single-cell transcriptomic analysis, we confirmed that GOT2 was down-regulated in cancer stem cells compared with HCC cells. Our study promotes the understanding of the metabolic heterogeneity in HCC which should be considered when developing personalized therapies.

Metabolic reprogramming drives HCC tumorigeneses and equips HCC with rapid progression ability, which also creates a vulnerability and may be potently exploited [49]. Studies keep focusing on the abnormal metabolism mechanisms and their therapeutic potential, concerning glucose, fatty acids and amino acids metabolism. Increased glutamine catabolism and absorbance from extracellular environment are critical features of HCC, and therapies based on which have been proposed as potential strategies for HCC treatment [50]. V-9302 is a small molecule antagonist of transmembrane glutamine flux which selectively and potently inhibits ASCT2-mediated glutamine uptake. V-9302 was proved to inhibit HCC growth both in vitro and in vivo, and sensitizes glutamine-dependent HCC cells to glutaminase inhibitor CB-839 treatment [51, 52]. Our study found that HCC patients with higher ASCT2 expression have worse prognosis. This indicates us with new insights on personalized medication. Patients with higher asparagine and glutamine metabolism may benefit extra from the combined treatment of ASCT2 inhibitor and first-line therapy. Notably, our study found that HCC patients with higher asparagine metabolism level have worse prognoses. Among the asparagine metabolism genes, GOT2 downregulation may be a critical factor that led to drug resistance and poor prognosis of HCC. The underlying mechanism may involve regulation of DDR and immune microenvironment. However, our study has limitations. In this study, we did bioinformatic exploration, yet not testify our findings in large scale clinical research or in biological research. The driving metabolic mechanism of HCC initiation and progression remain largely unknown and are under further investigation.

Cancer cells are characterized with alterations in DDR pathway and metabolism reprograming [53]. The links between these two fundamental processes have yet been clearly understood. The DNA reparation process relies on the cellular nucleotide level. Amino acids including glutamine and aspartate are essential for de novo nucleotide synthesis, thus exerting influence on the nucleotide availability, DNA replication and reparation. Glutamine is essential for inosine monophosphate synthesis, while aspartate is essential for the synthesis of pyrimidines [54, 55]. Previous studies showed that glioblastoma cells increase the glutamine synthesis by driving the αKG out of the TCA cycle, thus promoting the de novo purine synthesis [56]. This is in accordance with our study that the HCC subgroup with more glutamine intake and less entrance of αKG into TCA cycle has more active DNA reparation as revealed by higher expression of DDR molecules. The DDR pathway also contributes to the tumor metabolism reprogramming. The core molecule of DDR, p53, also plays roles in the metabolic reprogramming. p53 inhibits glycolysis through inhibiting the transcriptional activation of the TP53-induced glycolysis and apoptosis regulator (TIGAR) protein [57]. Moreover, p53 can also bind to G6PD and prevent its active dimer formation, which inhibits the pentose phosphate pathway (PPP) process [58]. Our study finds that in HCC cells with higher level of asparagine and glutamine, the DDR pathway is more active, with increased expression of DDR core molecules. Given that the first-line therapy sorafenib has no therapeutic difference over these two HCC subgroups, DDR inhibitors could be introduced into treatment to improve the prognosis of patient with relatively over-activated DDR pathway.

The tumor immune microenvironment (TME) plays a role in metabolic plasticity, and vice versa. The tumor metabolic reprogramming regulates the differentiation and activation of Treg cells in TME. Treg cells in the TME of HCC contribute to the resistance to immunotherapy [59]. As tumor cells depleted the glutamine in TME, glutamine deficiency impairs the differentiation ability of T cell toward Th1, Th2, and Th17 cells, but have a less effect on Treg cells [60]. Accordingly, the decreased intracellular αKG, caused by the limited availability of extracellular glutamine, also promotes the generation of Treg cells rather than Th1 cells [61]. Amino acid availability restriction in the TME suppresses the antitumor activity of T effector cells, due to increased infiltration of Treg cells. Moreover, Tregs are more flexible in energy resource intake, allowing them to survive relatively harsh conditions in the TME [62]. In addition, the immune checkpoint signals may also regulate metabolic activity of tumor and immune cells [63]. Taken together, Treg cells gain advantage in the TME by its metabolic adaptability. Our study firstly found that Treg cells are more enriched in the TME of HCC patients with higher level of asparagine metabolism. Moreover, GOT2 gene expression is closely related with the Treg infiltration. The down-regulated GOT2 may be responsible for the increased Treg infiltration and the worse prognosis of HCC subgroup with higher level of asparagine metabolism. The underlying mechanism demands our further exploration.

In conclusion, we construct and validate an asparagine metabolism-based prognostic signature of HCC. We also highlight the changes in energy metabolism, DDR pathway activation, and TME composition in the two HCC subtypes. Moreover, we propose the GOT2 gene as a potential prognostic and treatment targeting biomarker of HCC. These findings may benefit the personalized treatment for HCC patients.

## Supplementary Information

**Additional file 1. Fig. S1**: The interaction between asparagine metabolism gene set.

**Additional file 2. Fig. S2**: Scheme of high and low asparagine metabolism HCC subgroups.

**Additional file 3: Fig. S3**: The expression of ICI response prediction gene sets in high- and low- asparagine metabolism subgroups.

**Additional file 4. Table S1**: Clinical information of high- and low- asparagine metabolism HCC subgroups in TCGA.

**Additional file 5. Table S2**: Clinical information of high- and low- GOT2 expression HCC subgroups in TCGA.

**Additional file 6. Table S3**: Six established gene sets for predicting the patients' response to ICI therapy.

## Data Availability

The datasets generated and/or analyzed during the current study are available in the TCGA (https://portal.gdc.cancer.gov/), GEO (https://www.ncbi.nlm.nih.gov/geo/query/acc.cgi?acc=GSE84598, https://www.ncbi.nlm.nih.gov/geo/query/acc.cgi?acc=GSM3064824), and ICGC (https://icgc.org/) repository.

## Abbreviations

ACC: Adrenocortical carcinoma
BLCA: Bladder urothelial carcinoma
BRCA: Breast invasive carcinoma
CESC: Cervical squamous cell carcinoma and endocervical adenocarcinoma
CHOL: Cholangiocarcinoma
COAD: Colon adenocarcinoma
DLBC: Lymphoid neoplasm diffuses large b-cell lymphoma
ESCA: Esophageal carcinoma
STES: Stomach and esophageal carcinoma
GBM: Glioblastoma multiforme
HNSC: Head and neck squamous cell carcinoma
KICH: Kidney chromophobe
KIRC: Kidney renal clear cell carcinoma
KIRP: Kidney renal papillary cell carcinoma
LGG: Brain lower grade glioma
LIHC/HCC: Liver hepatocellular carcinoma/hepatocellular carcinoma
LUAD: Lung adenocarcinoma
LUSC: Lung squamous cell carcinoma
MESO: Mesothelioma
OV: Ovarian serous cystadenocarcinoma
PAAD or PADD: Pancreatic adenocarcinoma
PCPG: Pheochromocytoma and paraganglioma
PRAD: Prostate adenocarcinoma
READ: Rectum adenocarcinoma
SARC: Sarcoma
SKCM: Skin cutaneous melanoma
STAD: Stomach adenocarcinoma
TGCT: Testicular germ cell tumors
THYM: Thymoma
THCA: Thyroid carcinoma
UCS: Uterine carcinosarcoma
UCEC: Uterine corpus endometrial carcinoma
UVM: Uveal melanoma
TCGA: The Cancer Genome Atlas
ICGC: International Cancer Genome Consortium
GEO: Gene Expression Omnibus
GO: Gene Ontology
KEGG: Kyoto Encyclopedia of Genes and Genomes
GSEA: Gene Set Enrichment Analysis
Tregs: T regulatory cells
PD-1/PD-L1: Programmed cell death/-ligand 1
TME: Tumor microenvironment
CTLA4: Cytotoxic T lymphocyte-associated antigen 4
LAG3: Lymphocyte-activation gene 3
TIM3: T cell immunoglobulin and mucin domain containing-3
VEGF: Vascular endothelial growth factor
DEGs: Differentially expressed genes
GDC: The Genomic Data Commons data portal
OS: Overall survival
PFI: Progression-free interval
DSS: Disease-specific survival
RFS: Recurrence-free survival
PARP: Poly ADP-ribose polymerase inhibitors
DDR: DNA damage response
ICB: Immune checkpoint blockers
TCA: Tricarboxylic acid
ASNS: Asparagine synthetase
Gln: Glutamine
Glu: Glutamate
Asn: Asparagine
Asp: Aspartate
αKG: α-Ketoglutaric acid
scRNA-Seq: Single-cell RNA sequencing
TIGAR: TP53-induced glycolysis and apoptosis regulator
PPP: Pentose phosphate pathway
