# Supplementary figures and images for: An asparagine metabolism-based classification reveals the metabolic and immune heterogeneity of hepatocellular carcinoma
Source: BMC Med Genomics. 2022 Oct 25;15:222. doi: 10.1186/s12920-022-01380-z (PMC9594908; doi:10.1186/s12920-022-01380-z)

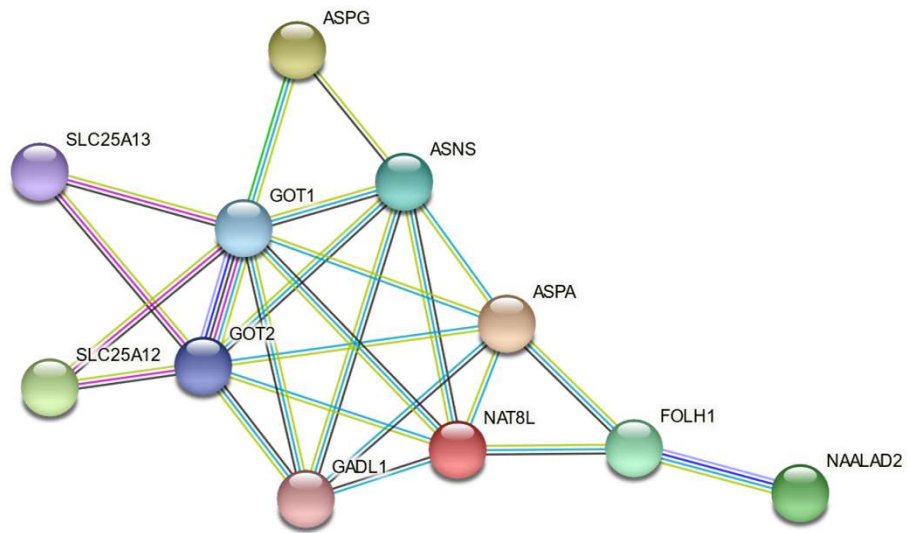

Supplementary Figure 1. The interaction between asparagine metabolism gene set.

Supplement: Supplementary file 1 — Additional file 1. Fig. S1: The interaction between asparagine metabolism gene set. [file 12920_2022_1380_MOESM1_ESM.pdf]
