# Supplementary material for: An asparagine metabolism-based classification reveals the metabolic and immune heterogeneity of hepatocellular carcinoma
Source: BMC Med Genomics. 2022 Oct 25;15:222. doi: 10.1186/s12920-022-01380-z (PMC9594908; doi:10.1186/s12920-022-01380-z)
Supplement: Supplementary file 2 — Additional file 2. Fig. S2: Scheme of high and low asparagine metabolism HCC subgroups. [file 12920_2022_1380_MOESM2_ESM.pdf]

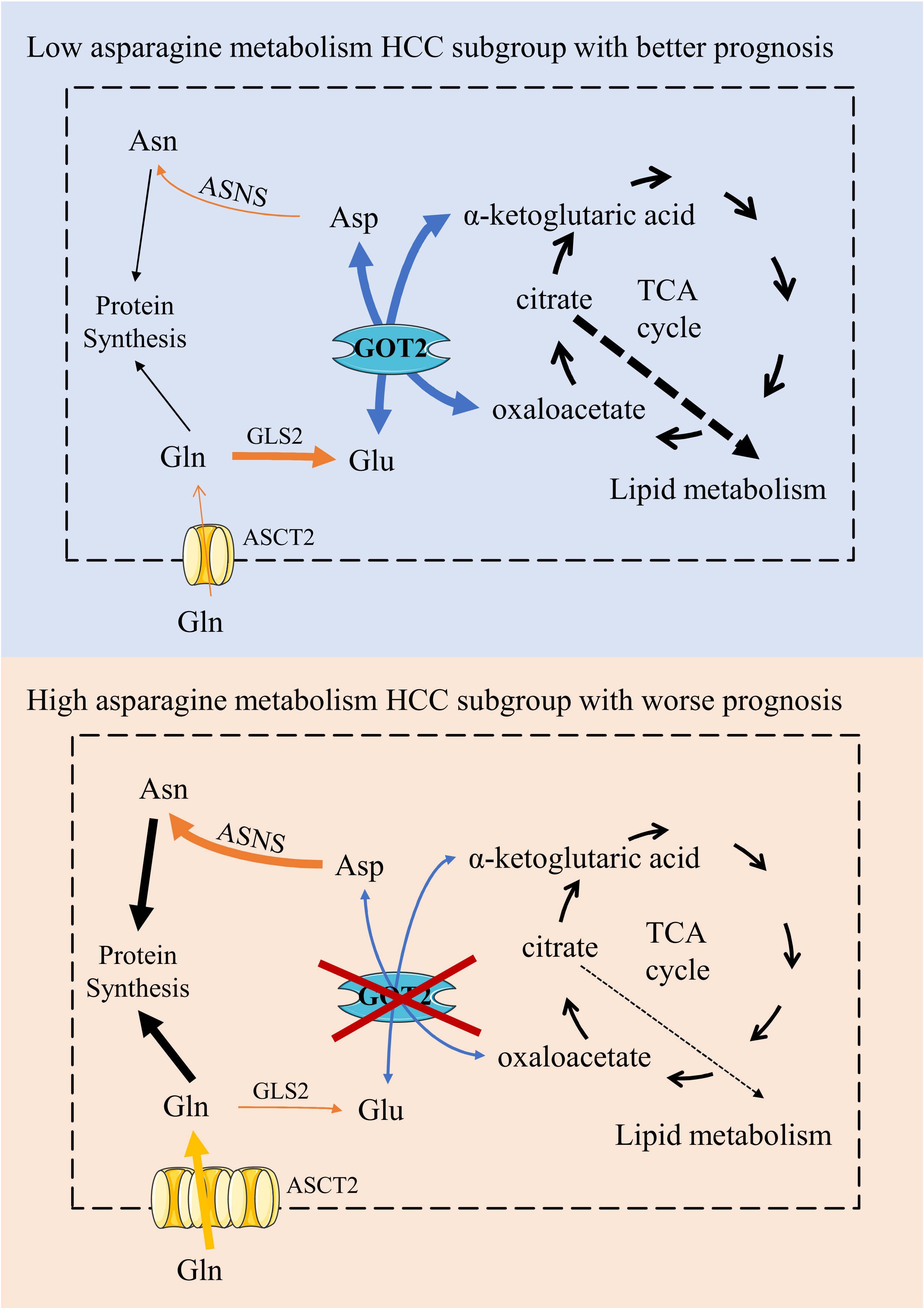

Supplementary Figure 2. Scheme of high and low asparagine metabolism HCC subgroups. GOT2: glutamic-oxaloacetic transaminase 2; Gln: Glutamine; Glu: glutamate; Asn: asparagine; Asp: aspartate; TCA: tricarboxylic acid; ASNS: asparagine synthetase; GLS2: glutaminase 2; ASCT2: Alanine, Serine, Cysteine Transporter 2
