# Supplementary material for: An asparagine metabolism-based classification reveals the metabolic and immune heterogeneity of hepatocellular carcinoma
Source: BMC Med Genomics. 2022 Oct 25;15:222. doi: 10.1186/s12920-022-01380-z (PMC9594908; doi:10.1186/s12920-022-01380-z)
Supplement: Supplementary file 3 — Additional file 3: Fig. S3: The expression of ICI response prediction gene sets in high- and low- asparagine metabolism subgroups. [file 12920_2022_1380_MOESM3_ESM.pdf]

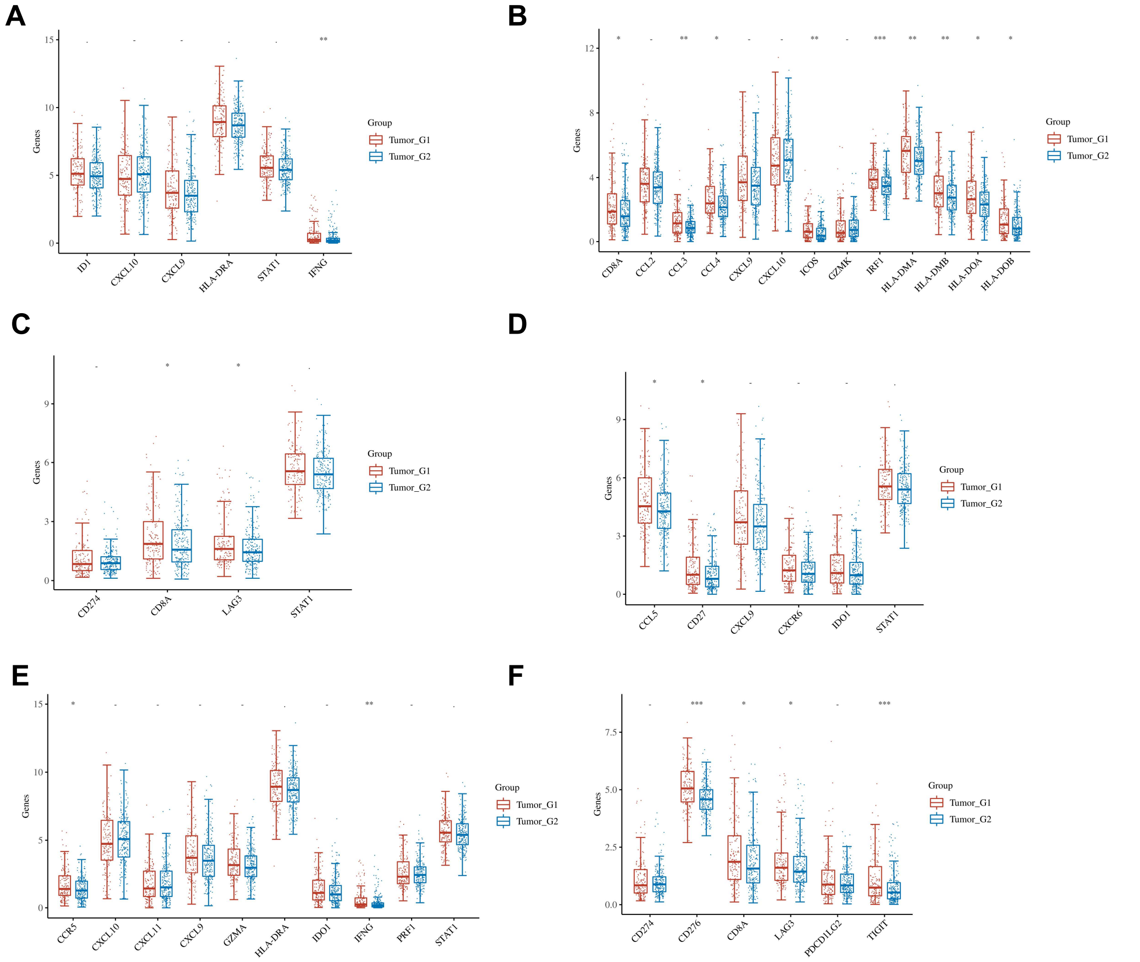

Supplementary Figure 3. The expression of ICI response prediction gene sets in high- and low- asparagine metabolism subgroups. A. 6-Gene Interferon Gamma. B. Gajewski 13-Gene Inflammatory. C. Inflammatory D. Interferon Gamma Biology. E. Ribas 10-Gene Interferon Gamma. F. T-cell Exhaustion. Tumor_G1 = high asparagine metabolism HCC group; Tumor_ G2 = low asparagine metabolism HCC group.
