# Supplementary material for: An asparagine metabolism-based classification reveals the metabolic and immune heterogeneity of hepatocellular carcinoma
Source: BMC Med Genomics. 2022 Oct 25;15:222. doi: 10.1186/s12920-022-01380-z (PMC9594908; doi:10.1186/s12920-022-01380-z)
Supplement: Supplementary file 4 — Additional file 4. Table S1: Clinical information of high- and low- asparagine metabolism HCC subgroups in TCGA. [file 12920_2022_1380_MOESM4_ESM.docx]

Supplementary Table 1. Clinical information of high- and low- asparagine metabolism HCC subgroups in TCGA

|  | **Characteristic** | **High Asn**  **metabolism group** | **Low Asn metabolism group** | **P_value** |
| --- | --- | --- | --- | --- |
| Status | Alive | 88 | 153 |  |
|  | Dead | 59 | 71 | 0.12 |
| Age | Mean (SD) | 58.8 (13.4) | 59.9 (13.6) |  |
|  | Median [MIN, MAX] | 59.5 [16,85] | 62 [17,90] | 0.457 |
| Gender | FEMALE | 54 | 67 |  |
|  | MALE | 93 | 157 | 0.208 |
| Race | AMERICAN INDIAN | 1 | 1 |  |
|  | ASIAN | 74 | 84 |  |
|  | BLACK | 7 | 10 |  |
|  | WHITE | 61 | 123 | 0.08 |
| pT_stage | T1 | 52 | 129 |  |
|  | T2 | 48 | 44 |  |
|  | T2b | 1 |  |  |
|  | T3 | 22 | 23 |  |
|  | T3a | 17 | 12 |  |
|  | T3b | 3 | 3 |  |
|  | T4 | 4 | 9 |  |
|  | T2a |  | 1 |  |
|  | TX |  | 1 | 0.001 |
| pN_stage | N0 | 113 | 139 |  |
|  | N1 | 1 | 3 |  |
|  | NX | 33 | 81 | 0.013 |
| pM_stage | M0 | 115 | 151 |  |
|  | M1 | 2 | 2 |  |
|  | MX | 30 | 71 | 0.055 |
| pTNM_stage | I | 50 | 121 |  |
|  | II | 42 | 44 |  |
|  | III | 1 | 2 |  |
|  | IIIA | 37 | 28 |  |
|  | IIIB | 4 | 4 |  |
|  | IIIC | 3 | 6 |  |
|  | IV | 1 | 1 |  |
|  | IVB | 1 | 1 |  |
|  | IVA |  | 1 | 0.007 |
| Grade | G1 | 14 | 41 |  |
|  | G2 | 64 | 113 |  |
|  | G3 | 61 | 61 |  |
|  | G4 | 7 | 5 | 0.005 |
| new_tumor_event_type | Primary | 5 | 5 |  |
|  | Recurrence | 68 | 95 | 0.853 |
| Radiation_therapy | Non-radiation | 88 | 152 |  |
|  | Radiation | 1 | 3 | 1 |
| History_of_neoadjuvant_treatment | No neoadjuvant | 147 | 222 |  |
|  | Neoadjuvant |  | 2 |  |
| Therapy_type | Ancillary | 1 |  |  |
|  | Chemotherapy | 9 | 20 |  |
|  | Chemotherapy:Hormone Therapy | 1 |  |  |
|  | Chemotherapy:Hormone Therapy:Other. specify in notes | 1 |  |  |
|  | Chemotherapy:Targeted Molecular therapy | 1 | 1 |  |
|  | Targeted Molecular therapy | 2 | 3 |  |
|  | Other. specify in notes |  | 1 | 0.811 |
