# Supplementary material for: An asparagine metabolism-based classification reveals the metabolic and immune heterogeneity of hepatocellular carcinoma
Source: BMC Med Genomics. 2022 Oct 25;15:222. doi: 10.1186/s12920-022-01380-z (PMC9594908; doi:10.1186/s12920-022-01380-z)
Supplement: Supplementary file 5 — Additional file 5. Table S2: Clinical information of high- and low- GOT2 expression HCC subgroups in TCGA. [file 12920_2022_1380_MOESM5_ESM.docx]

Supplementary Table 2. Clinical information of high- and low- GOT2 expression HCC subgroups in TCGA

| Characteristic | Low expression of GOT2 | High expression of GOT2 | p |
| --- | --- | --- | --- |
| n | 187 | 187 |  |
| T stage, n (%) |  |  | 0.011 |
| T1 | 79 (21.3%) | 104 (28%) |  |
| T2 | 58 (15.6%) | 37 (10%) |  |
| T3 | 45 (12.1%) | 35 (9.4%) |  |
| T4 | 4 (1.1%) | 9 (2.4%) |  |
| N stage, n (%) |  |  | 1.000 |
| N0 | 129 (50%) | 125 (48.4%) |  |
| N1 | 2 (0.8%) | 2 (0.8%) |  |
| M stage, n (%) |  |  | 1.000 |
| M0 | 141 (51.8%) | 127 (46.7%) |  |
| M1 | 2 (0.7%) | 2 (0.7%) |  |
| Gender, n (%) |  |  | 0.377 |
| Female | 65 (17.4%) | 56 (15%) |  |
| Male | 122 (32.6%) | 131 (35%) |  |
| Race, n (%) |  |  | 0.815 |
| Asian | 77 (21.3%) | 83 (22.9%) |  |
| Black or African American | 8 (2.2%) | 9 (2.5%) |  |
| White | 95 (26.2%) | 90 (24.9%) |  |
| Age, n (%) |  |  | 0.961 |
| <=60 | 89 (23.9%) | 88 (23.6%) |  |
| >60 | 97 (26%) | 99 (26.5%) |  |
| Tumor status, n (%) |  |  | 0.122 |
| Tumor free | 93 (26.2%) | 109 (30.7%) |  |
| With tumor | 84 (23.7%) | 69 (19.4%) |  |
| Prothrombin time, n (%) |  |  | 0.281 |
| <=4 | 94 (31.6%) | 114 (38.4%) |  |
| >4 | 47 (15.8%) | 42 (14.1%) |  |
| Albumin(g/dl), n (%) |  |  | 0.421 |
| <3.5 | 29 (9.7%) | 40 (13.3%) |  |
| >=3.5 | 112 (37.3%) | 119 (39.7%) |  |
| Child-Pugh grade, n (%) |  |  | 0.213 |
| A | 105 (43.6%) | 114 (47.3%) |  |
| B | 13 (5.4%) | 8 (3.3%) |  |
| C | 1 (0.4%) | 0 (0%) |  |
| Fibrosis ishak score, n (%) |  |  | 0.066 |
| 0 | 32 (14.9%) | 43 (20%) |  |
| 1/2 | 21 (9.8%) | 10 (4.7%) |  |
| 3/4 | 15 (7%) | 13 (6%) |  |
| 5/6 | 34 (15.8%) | 47 (21.9%) |  |
| Age, median (IQR) | 61 (51, 69) | 61 (54, 68) | 0.598 |
